# Trigger factors in primary headaches subtypes: a cross-sectional study from a tertiary centre in Greece

**DOI:** 10.1186/s13104-015-1390-7

**Published:** 2015-09-01

**Authors:** Panagiotis Iliopoulos, Dimitris Damigos, Elli Kerezoudi, Georgia Limpitaki, Michael Xifaras, Dionysoula Skiada, Aikaterini Tsagkovits, Petros Skapinakis

**Affiliations:** Department of Neurology, Pammakaristos Hospital, Iakovaton 43, Athens, 11142 Greece; Department of Psychiatry, University of Ioannina School of Medicine, 45110 Ioannina, Greece; Department of Neurosurgery, University Hospital of Ioannina, 45110 Ioannina, Greece; Department of Otorhinolaryngology, Pammakaristos Hospital, 11142 Athens, Greece

**Keywords:** Trigger factors, Migraine, Aura, Tension type headache

## Abstract

**Background:**

Previous studies have shown that common headache triggers contribute to the onset of headache attacks on predisposed individuals and are considered important in the prevention of headache. The aim of this study was to compare the different characteristics of triggers among the most common primary headache subtypes (migraine without aura, migraine with aura and tension type headache).

**Methods:**

A total of 116 headache patients of the neurology outpatient department of a tertiary hospital in Athens were selected according to the criteria of the International Classification of Headaches—3nd Edition Beta. Patients were interviewed using a questionnaire that contained 35 potential trigger factors.

**Results:**

The findings showed that migraine and tension-type headache patients report multiple triggers, on a frequent but variable basis. The most frequent triggers reported by all subjects were stressful life events followed by intense emotions. The same applies to both genders, as well as the three headache subgroups. Patients suffering from migraine with aura reported the highest mean number of trigger per person and the highest frequency in almost all the trigger categories. Furthermore, patients with migraine with aura were more likely to report the following triggers: oversleeping, premenstrual period, stressful life events, hot/cold weather, relaxation after stress, menstruation, wind, intense emotions, shining, hunger and bright sunlight. These associations were mostly independent of the sociodemographic characteristics and the presence of anxiety or depressive symptoms.

**Conclusion:**

The sensitivity to trigger factors should be considered by both clinicians and headache sufferers.

## Background

Headache disorders, especially the primary types, are considered as major global health problems due to their high prevalence, their almost life-long duration and their substantial disability burden upon the sufferers [1]. The most common primary headaches, tension-type headache and migraine are ranked as the second and third most common complications (“sequelae”) of the diseases covered by the Global Burden of Disease study for all ages in 2010 [2] and migraine itself is called “the seventh disabler’’ nowadays [3].

Despite the progress in the prevention and acute drug treatment of headaches, the recognition and management of the potential trigger factors remains an important key element for the successful clinical approach of headache patients [4]. The term ‘headache trigger’ is used to describe any stimulus that alone or in combination, contributes to the onset of a headache attack on predisposed individuals [5, 6]. Current suggestions of how triggers influence nociceptive pathways and induce headache attacks are still lacking strong experimental evidence [7].

Several studies on migraine and tension type headache indicate a long list of precipitating factors that are commonly reported by people suffering from recurrent headaches. A review of the literature demonstrated stress, skipping meals, sleep deprivation and weather changes as the most frequently mentioned triggers [8–10], even though factors such as fatigue, food, menstrual cycle, sunlight are consistently cited with various prevalence proportions [11–13], possibly due to different socio-cultural characteristics of the samples used.

Previous studies primarily focus on analyzing a specific precipitating factor of a headache type. Some studies have tried to investigate differences in the distribution of trigger factors between migraine and tension type headache (TTH) [10, 14–16]. Despite the ever-increasing suggestion that migraine subtypes, migraine without aura (MoA) and migraine with aura (MA), are separate entities due to their different clinical and pathophysiological background [17, 18], only a few studies investigated the variations among MoA, MA and TTH triggers [19–21]. Their conclusions reveal some degree of uniformity. Nonetheless, some discrepancies do exist and they consequently stimulated our research in this area.

The main aim of the study was to investigate the trigger factors of migraine with aura, migraine without aura and tension-type headache and to compare their differences in prevalence and likelihood of occurrence among these primary headaches. Our secondary aim was to investigate whether these associations were independent of the patients’ sociodemographic characteristics and the presence of anxiety and depressive symptoms.

## Methods

### Description of the study population

The present study was conducted between February 2013 and February 2014 at the neurology outpatient department of a tertiary hospital of Athens, Greece (General Hospital ‘Pammakaristos’). The hospital mostly caters to middle and low economic class people residing in the city center. Patients, who were referred to the hospital or were seen as outpatients with a chief complaint of headache, were recruited.

### Description of the procedure

A neurological examination and an extensive face-to-face interview was carried out by the researchers using a designated questionnaire pertaining biographical data, lifestyle habits, medical history and clinical headache characteristics (disorder history, frequency, attack duration, presence of aura, intensity, associated symptoms such as nausea, vomiting, photophobia, phonophobia, unilateral tearing or nasal congestion)required to establish a diagnosis. The diagnoses were made in accordance with the criteria of the International Classification of Headaches—3nd Edition Beta (ICHD—III beta) [24]. Subsequently, the participants were asked to grade 35 potential trigger factors, which were selected from previous studies, on a 4-point scale based on their likelihood of provoking an attack. The selected list of trigger factors included: dietary factors, such as fasting, skipping meals, consumption of dairy products, chocolate, alcohol, coffee, fruits/vegetables, fatty meals, spicy foods, cold/frozen food; coffee deprivation hormonal factors (menstruation, premenstrual period); sleep habits, such as oversleep, lack of sleep, changes in sleep hours; environmental factors, such as weather changes, hot/cold weather, rain, wind, bright sunlight, noises, shining, pollution, cigarette smoke, odors; stress levels caused by stressful life events, intense emotions (excessive sadness or happiness) relaxation after stress; participation in several activities, such as physical activity, sexual activity, fatigue, relaxation and travelling. Finally, the subjects completed the Hospital Anxiety and Depression Scale (HADS) [25] in order to determine the levels of anxiety and depressive symptoms that each subject was experiencing the past few weeks. We used the 75th percentile in each subscale (anxiety–depression) in order to define patients with high anxiety and depressive symptoms. We used these binary variables in our analysis.

### Exclusion criteria

Subjects excluded from the study were those: (a) below the age of 18 and over 70-years-old, (b) having a headache disorder for less than 12 months, (c) with an additional type of primary or secondary headache (trauma, underlying neurologic impairment), especially headache caused by medication overuse, (d) who were cognitively not capable of completing the questionnaire, (e) with serious psychiatric disease, (f) who declined to participate in the study.

### Statistical analysis

Data were analyzed using the Statistical package for Social Sciences (SPSS) 17.0 for Windows software. The answers in the section of trigger factors were dichotomized, with the effect that the ‘never/rarely’ replies were perceived as negative, accordingly, the ‘often/always’ ones as positive for the presence or absence of a trigger factor. Descriptive statistics were used to analyze the demographic, as well as, lifestyle characteristics (frequency, mean, median, standard deviation). Pearson’s Chi square and Fisher’s Exact were used to evaluate the correlation between the types of headache and the trigger factors (crosstabs procedure). The sum of triggers per patient was used as an independent variable. To investigate the association between the types of headache and the trigger factors, we used multinomial logistic regression from which estimated adjusted odds ratios with tension type headache as a baseline category. Adjustments were made for the sociodemographic characteristics and the presence of anxiety or depressive symptoms.

### Ethical consideration

The study protocol was approved by the Ethics Committee of Pammakaristos Hospital. All patients gave their written informed consent for participation in the study.

## Results

### Demographic characteristics

Out of the 116 patients included in the study, 93 (80.2 %) were female and 23 (19.8 %) male. The majority (56 %) of the sample were between 30 and 49 years of age. Overall mean age was 40.7 [standard deviation (SD) 13.0] years, while the mean age of female subjects was 40.5 (SD 13.1) years and 41.6 (SD 12.7) of male subjects, respectively. As can be seen in Table 1, 29.3 % were single, 57.8 % married, 11.2 % divorced, and 1.7 % widowed. The average number of children per patient was 1.33, whereas 40.5 % of the sample had no children. Almost half (44.8 %) of the patients were higher education graduates and only 3.4 % lack mastery of basic educational skills. Finally, 58.6 % had a job and 41.4 were unemployed. No statistically significant variation was detected among MoA, MA and TTH (Table 1).

**Table 1:** Sociodemographic data and clinical characteristics in three study groups

	Total (N = 116)	MoA (N = 47)	MA (N = 41)	TTH (N = 28)	p value
Sex—no. (%)					
Men	23 (19.8)	11(23.4)	4 (9.8)	8 (28.6)	0.110
Women	93 (80.2)	36 (76.6)	37 (90.2)	20 (71.4)	
Age group—no. (%)					
18–29	20 (17.2)	8 (17.0)	7 (17.1)	5 (17.9)	0.923
30–49	66 (56.9)	28 (59.6)	24 (58.5)	14 (50.0)	
50–69	30 (25.9)	11 (23.4)	10 (24.4)	9 (32.1)	
Marital status—no. (%)					
Single	34 (29.3)	14 (29.8)	13 (31.7)	7 (25.0)	0.989
Married	67 (57.8)	26 (55.3)	23 (56.1)	18 (64.3)	
Divorced	13 (11.2)	6 (12.8)	4 (9.8)	3 (10.7)	
Widowed	2 (1.7)	1 (2.1)	1 (2.4)	0 (0.0)	
Education level—no. (%)					
Without basic	4 (3.4)	1(2.1)	2 (4.9)	1 (3.6)	0.576
Primary school	13 (11.2)	8 (17.0)	3 (7.3)	2 (7.1)	
Secondary school	6 (5.2)	2 (4.3)	2 (4.9)	2 (7.1)	
High school	41 (35.3)	19 (40.4)	11 (26.8)	11 (39.3)	
Higher education	52 (44.8)	17 (36.2)	23 (56.1)	12 (42.8)	
Employment status—no. (%)					
Employed	68 (58.6)	31 (66.0)	24 (58.5)	13 (46.4)	0.252
Unemployed	48 (41.4)	16 (34.0)	17 (41.5)	15 (53.6)	
Physical activity—mean (SD) (h)	2.0 (2.7)	1.6 (2.3)	2.2 (2.7)	2.3 (3.4)	0.607
Sleep duration—mean (SD) (h)	6.9 (1.2)	7.1 (1.3)	6.9 (1.3)	6.6 (1.1)	0.101
Time until sleep occurrence—mean (SD) (min)	29.3 (26.0)	26.5 (21.5)	29.0 (31.3)	34.3 (24.8)	0.243
Coffee—mean (SD) (cups/day)	1.4 (1.0)	1.4 (1.0)	1.5 (1.0)	1.3 (1.0)	0.740
Alcohol—mean (SD) (drinks/day)	0.2 (0.5)	0.1 (0.5)	0.2 (0.4)	0.3 (0.5)	0.135
Smoke—mean (SD) (cigarettes/day)	6.9 (10.9)	7.8 (11.6)	5.7 (9.7)	7.3 (11.7)	0.656
Disorder duration—mean (SD) (years)	13.1 (11.9)	14.1 (12.1)	16.1 (12.1)	6.9 (9.1)	<0.001
Attacks frequency—mean (SD) (number/month)	5.7 (4.9)	3.9 (3.2)	3.5 (2.3)	11.6 (5.2)	<0.001
Attack duration—mean (SD) (h)	16.3 (16.6)	19.7 (17.5)	18.6 (17.5)	7.1 (9.2)	<0.001
Attack intensity—mean (SD) (1–10)	7.6 (1.7)	8.1 (1.2)	7.9 (1.6)	6.4 (1.9)	<0.001
Disability for work—mean (SD) (1–3)	2.2 (0.8)	2.2 (0.8)	2.4 (0.7)	1.7 (0.8)	0.003
Disability for everyday activities—mean(SD) (1–3)	2.1 (0.8)	2.2 (0.9)	2.4 (0.7)	1.6 (0.7)	<0.001
Anxiety score—mean (SD) (1–21)	7.5 (4.2)	7.3 (3.8)	8.0 (4.3)	7.4 (4.9)	0.798
Depression score—mean (SD) (1–21)	6.0 (3.9)	6.6 (3.9)	5.3 (3.8)	6.1 (4.0)	0.213

MoA, MA, TTH denote migraine without aura, migraine with aura and tension-type headache, respectively

### Lifestyle characteristics

The lifestyle habits of our sample were recorded in order to determine whether headaches are affected by these habits. The mean duration of physical exercise was 2.0 (SD 2.7) h per week, the mean sleep duration was 6.9 (SD 1.2) h and the mean time until sleep occurrence was 29.3 (SD 26.0) min. The results showed that our subjects consumed 1.4 (SD 1.0) cups of coffee, drank a small amount of alcohol, and smoked an average of 6.9 (SD 10.9) cigarettes per day. None of the above parameters differed on a statistically significant basis in the three study groups (Table 1).

### Headache characteristics

According to ICD-III beta criteria, 47 (40.5 %) of the subjects were diagnosed with migraine without aura (MoA), 41 (35.3 %) with migraine with aura (MA) and 28 (24.1 %) with tension-type headache (TTH). The median duration of the headache history was 10 years. The median frequency of attacks per month was 3 and the median attack duration was 9.5 h. In a scale from 1 to 10, patients recorded 8 as the median intensity of their headache attacks. They mentioned that the attacks result in moderate to severe disability to work and engage in everyday activities. All headache characteristics were statistically significant different in the three study groups, as shown in Table 1, using non-parametric Kruskal–Wallis test.

### Anxiety and depressive symptoms

HADS data analysis showed that 25.9 % of the sample recorded high levels of anxiety symptoms while 14.7 % had depressive symptoms, without statistically significant differences among MoA, MA and TTH. Women had more anxiety (p = 0.037) and depressive (p = 0.022) symptoms compared to men (Table 1).

### Trigger characteristics

#### Comparison of the sum of triggers per subject

The majority of the patients reported multiple precipitating factors. The mean number of triggers in our sample was 9.8 (SD 5.4; median 9). Only 2 patients (1.72 %) did not report any trigger. Women reported more triggers (mean 10.2; SD 5.3; median 9) than men (mean 8.04; SD 5.2; median 7), without any statistically significant difference (p = 0.053). Subjects with MA reported considerably more triggers (p < 0.001) than patients with MoA and TTH. The mean number of triggers in headache groups was in the MoA group 8.1 (SD 4.3; median 8), in the MA group 12.6 (SD 5.4; median 12) and in the TTH group 8.4 (SD 5.4; median 7).

#### Likelihood of trigger factor occurrence

**Fig. 1:**
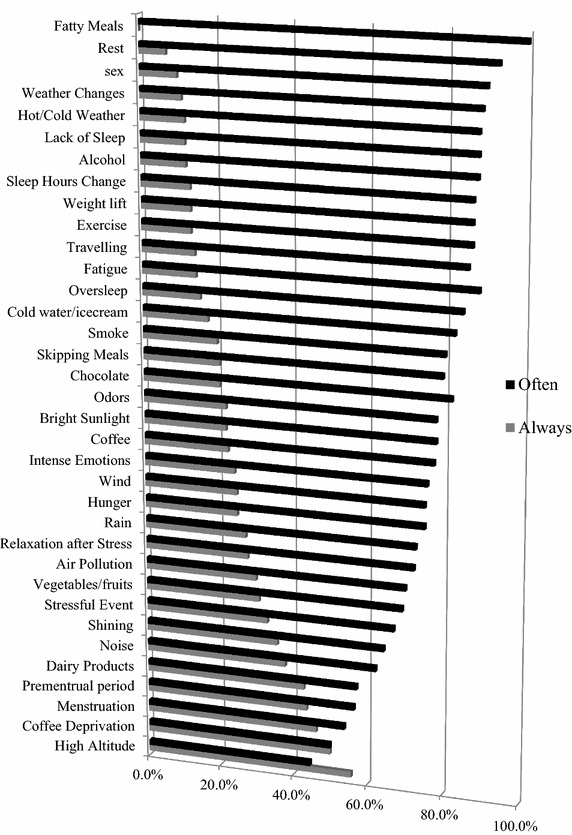
‘Often’ (*black*) vs. ‘Always’ (*grey*) responds in trigger factors occurrence (total sample)

The vast majority of patients tended to report a frequent, not a constant occurrence of factors triggering headache attacks. In 33 out of 35 trigger categories, subjects answered ‘often’ more frequently than ‘always’, with the exception of coffee deprivation and high altitude factors. A statistically significant difference among the three study groups was observed only in chocolate (p = 0.021) and in air pollution (p = 0.027). In total, the patients cited 76 % ‘often’ and 24 % ‘always’ (Fig. 1).

#### Comparison of trigger factors between genders

The results concerning trigger factors within the total sample and comparison between genders are listed in Table 2. The most common triggers cited in this study were stressful life events (83.62 %), followed by intense emotions (70.69 %) overall, in both sexes and among the three study groups. More than half of the female subjects (55.91 %) reported menstruation as a potential trigger. Other factors subsequently registered were lack of sleep (50.86 %), fatigue (47.41 %), weather changes (46.55 %), premenstrual period in women (41.94 %) and odors (38.79 %). At the other extreme, fatty meals, sexual activity and high altitude were the least common triggers. The triggers did not differ significantly between genders with the exceptions of noises (p = 0.021) in women.

**Table 2:** Trigger factors distribution in genders

Trigger factors	Gender			p value
	Total (%)	Women (%)	Men (%)	
Stressful event	83.62	87.10	69.57	0.058
Intense emotions	70.69	76.34	47.83	0.009
Menstruation	55.91	55.91	NA	NA
Sleep deprivation	50.86	51.61	47.83	0.745
Fatigue	47.41	49.46	39.13	0.372
Weather changes	46.55	47.31	43.48	0.741
Premenstrual period	41.94	41.94	NA	NA
Odors	38.79	40.86	30.43	0.352
Hot/cold weather	36.21	35.48	39.13	0.746
Sleep hours changes	32.76	33.33	30.43	0.790
Noises	31.90	36.56	13.04	0.021
Hunger	31.03	30.11	34.78	0.667
Relaxation after stress	31.03	32.26	26.09	0.562
Traveling	30.17	27.96	39.13	0.304
Skipping meals	29.31	26.88	39.13	0.257
Alcohol	28.45	24.73	43.48	0.083
Oversleep	27.59	27.96	26.09	1.000
Coffee deprivation	25.86	24.73	30.43	0.600
Wind	24.14	20.43	39.13	0.071
Bright sunlight	23.28	24.73	17.39	0.444
Cigarette smoke	21.55	24.73	8.70	0.102
Rain	18.97	17.20	26.09	0.375
Chocolate	18.97	20.43	13.04	0.559
Coffee consumption	18.97	21.51	8.70	0.168
Air pollution	17.24	19.35	8.70	0.250
Cold drinks/ice cream	14.66	15.05	13.04	1.000
Physical activity	12.93	13.98	8.70	0.536
Weight lift	12.93	11.83	17.39	0.732
Rest	12.07	13.98	4.35	0.296
Dairy products	12.07	13.98	4.35	0.296
Shining	12.07	12.90	8.70	0.733
Vegetables	11.21	12.90	4.35	0.305
Fatty meals	8.62	9.68	4.35	0.478
Sexual activity	8.62	7.53	13.04	0.684
High altitude	7.76	8.60	4.35	0.686

*NA* not applicable

#### Comparison of trigger factors among MoA, MA and TTH

The patients with MA reported the highest frequency in almost all the trigger factors categories. Using Pearson’s Chi square tests, a statistically significant difference was recorded for oversleeping, premenstrual period, stressful life events, hot/cold weather, and relaxation after stress, menstruation, wind, intense emotions, shining, hunger, and bright sunlight, which were more commonly reported in MA. Higher frequencies with no statistically significant association emerged in TTH for physical activities, fatigue, weight lifting, and resting. Vegetables/fruits and cold water/ice cream were cited more often in MoA but no significant correlation was observed. The distribution of all the triggers and the differences among the three headache groups are presented in Table 3.

**Table 3:** Comparison of trigger factors frequencies among migraine without aura, migraine with aura and tension type headache

Trigger factors	Headache type			
	Migraine without aura (%)	Migraine with aura (%)	Tension type headache (%)	p value
Oversleep	17.02	46.34	17.86	0.004
Premenstrual period	27.66	51.22	17.86	0.008
Stressful life event	72.34	95.12	85.71	0.011
Hot/cold weather	25.53	53.66	28.57	0.015
Relaxation after stress	17.02	43.90	35.71	0.018
Menstruation	31.91	60.98	42.86	0.022
Wind	17.02	39.02	14.29	0.023
Intense emotions	57.45	82.93	75.00	0.027
Shining	8.51	21.95	3.57	0.043
Hunger	31.91	41.46	14.29	0.044
Bright sunlight	17.02	36.59	14.29	0.046
Alcohol	27.66	39.02	14.29	0.071
Cigarette smoke	14.89	34.15	14.29	0.057
Skipping meals	31.91	36.59	14.29	0.097
Rain	14.89	29.27	10.71	0.106
Noise	23.40	43.90	28.57	0.112
Air pollution	8.51	24.39	21.43	0.112
Coffee deprivation	17.02	34.15	28.57	0.167
High altitude	4.26	14.63	3.57	0.179
Odors	36.17	48.78	28.57	0.212
Sexual activity	6.38	14.63	3.57	0.217
Weather changes	38.30	56.10	46.43	0.247
Sleep hours changes	29.79	41.46	25.00	0.309
Physical activities	10.64	9.76	21.43	0.357
Fatigue	40.43	51.22	53.57	0.451
Traveling	31.91	34.15	21.43	0.484
Vegetables	14.89	9.76	7.14	0.609
Sleep deprivation	48.94	56.10	46.43	0.690
Fatty meals	6.38	12.20	7.14	0.698
Weight lift	10.64	12.20	17.86	0.728
Chocolate	17.02	21.95	17.86	0.831
Coffee	17.02	21.95	17.86	0.831
Dairy products	10.64	14.63	10.71	0.877
Rest	10.64	12.20	14.29	0.897
Cold water/ice cream	14.89	14.63	14.29	1.000

#### Trigger factors adjusted for sociodemographic data, anxiety and depressive symptoms

MA remained statistically significant associated with more triggers than TTH after being adjusted for sociodemographic data and anxiety/depressive symptoms [odds ratio (OR) 1.21 (95 % CI 1.06–1.37)]. Similarly, the majority of trigger factors did not seem to be affected by these factors except for the cases of shining and skipping meals (Table 4).

**Table 4:** Multinomial logistic regression analyses of trigger factors of MA and MoA vs. TTH (base category) adjusted with sociodemographic variables, anxiety and depressive symptoms (HADS)

Disorder	MA	MoA	MA	MoA
	Crude OR (95 %CI)	Crude OR (95 % CI)	Adjusted OR (95 % CI)	Adjusted OR (95 % CI)
*Trigger factors*				
Oversleep	3.97 (1.26–12.49)*	0.94 (0.28–3.23)	4.65 (1.32–16.33)*	1.00 (0.27–3.73)
Premenstrual period	4.83 (1.54–15.17)**	1.76 (0.55–5.61)	5.02 (1.26–19.98)*	2.18 (0.54–8.72)
Stressful life event	3.25 (0.55–19.12)	0.44 (0.18–1.50)	3.60 (0.55–23.41)	0.50 (0.13–1.97)
Hot/cold weather	2.89 (1.04–8.06)*	0.77 (0.30–2.45)	4.92 (1.31–18.57)*	0.81 (0.22–2.98)
Relaxation after stress	1.41 (0.52–3.79)	0.37 (0.13–1.09)	1.02 (0.31–3.32)	0.24 (0.07–0.89)
Menstruation	2.08 (0.78–5.53)	0.63 (0.24–1.65)	1.68 (0.47–5.95)	0.57 (0.16–2.00)
Wind	3.84 (1.12–13.14)*	1.23 (0.33–4.53)	10.26 (1.96–53.73)**	2.71 (0.52–14.08)
Intense emotions	1.62 (0.48–5.27)	0.45 (0.16–1.26)	1.38 (0.36–5.23)	0.48 (0.14–1.62)
Shining	7.59 (0.90–63.80)	2.51 (0.27–23.68)	18.21 (1.47–225.24)*	2.25 (0.18–28.86)
Hunger	4.25 (1.25–14.50)*	2.81 (0.83–9.56)	4.97 (1.24–19.87)*	3.11 (0.79–12.30)
Bright sunlight	3.46 (1.01–11.90)*	1.23 (0.33–4.53)	4.81 (1.21–19.22)*	1.35 (0.37–5.61)
Alcohol	3.84 (1.12–13.14)*	2.29 (0.67–7.90)	5.61 (1.42–22.11)*	2.52 (0.66–9.67)
Cigarette smoke	3.11 (0.90–10.75)	1.05 (0.28–3.97)	3.55 (0.84–15.00)	1.36 (0.30–6.15)
Skipping meals	3.46 (1.01–11.90)*	2.81 (0.83–9.56)	3.83 (0.98–15.06)	2.40 (0.63–9.18)
Rain	3.49 (0.87–13.62)	1.46 (0.45–6.17)	3.66 (0.77–17.33)	1.20 (0.25–5.84)
Noise	1.96 (0.70–5.46)	0.76 (0.26–2.21)	2.03 (0.64–6.43)	0.81 (0.25–2.64)
Air pollution	1.18 (0.37–3.73)	0.34 (0.09–1.33)	0.96 (0.27–3.46)	0.27 (0.06–1.20)
Coffee deprivation	1.29 (0.45–3.68)	0.51 (0.17–1.57)	1.29 (0.40–4.15)	0.53 (0.15–1.80)
High altitude	4.63 (0.52–40.77)	1.20 (0.10–13.87)	4.15 (0.38–45.44)	1.18 (0.08–17.14)
Odors	2.38 (0.85–6.62)	1.42 (0.51–3.90)	2.93 (0.89–9.65)	1.44 (0.45–4.63)
Sexual activity	4.63 (0.52–40.77)	1.84 (0.18–18.61)	5.90 (0.54–64.27)	1.33 (0.11–15.85)
Weather changes	1.47 (0.56–3.87)	0.72 (0.28–1.85)	1.66 (0.53–5.19)	0.75 (0.24–2.30)
Sleep hours changes	2.12 (0.74–6.11)	1.27 (0.44–3.67)	2.18 (0.69–6.86)	1.25 (0.40–3.89)
Physical activities	0.40 (0.10–1.56)	0.44 (0.12–1.59)	0.22 (0.07–1.06)	0.33 (0.07–1.46)
Fatigue	0.91 (0.34–2.38)	0.59 (0.23–1.51)	0.71 (0.23–2.18)	0.46 (0.15–1.36)
Traveling	1.90 (0.63–5.77)	1.72 (0.58–5.12)	2.21 (0.67–7.32)	2.02 (0.62–6.56)
Vegetables	1.40 (0.24–8.25)	2.28 (0.44–11.81)	1.27 (0.17–9.28)	1.87 (0.29–12.14)
Sleep deprivation	1.47 (0.56–3.87)	1.11 (0.43–2.82)	1.93 (0.64–5.83)	1.62 (0.55–4.76)
Fatty meals	1.80 (0.32–10.04)	0.89 (0.14–5.66)	1.73 (0.21–14.32)	0.52 (0.06–4.60)
Weight lift	0.64 (0.16–2.45)	0.55 (0.14–2.09)	0.52 (0.13–2.60)	0.48 (0.11–2.14)
Chocolate	1.29 (0.38–4.37)	0.94 (0.28–3.23)	0.95 (0.25–3.68)	0.52 (0.13–2.02)
Coffee	1.29 (0.38–4.37)	0.94 (0.28–3.23)	0.87 (0.21–3.61)	0.60 (0.14–2.53)
Dairy products	1.43 (0.32–6.26)	0.99 (0.22–4.51)	1.18 (0.24–5.79)	0.80 (0.15–4.19)
Rest	0.83 (0.20–3.42)	0.71 (0.18–2.92)	0.61 (0.12–3.18)	0.59 (0.11–3.02)
Cold water/ice cream	1.03 (0.26–4.03)	1.05 (0.26–4.04)	0.86 (0.17–4.30)	0.52 (0.11–2.45)

MA, MoA, TTH denote migraine with aura, migraine without aura and tension-type headache respectively

Sociodemographic variables: sex, age group, marital status, educational level, employment status

Level of significance: * <0.05 and ** <0.01

*HADS* Hospital Anxiety and Depression Scale, *Crude OR* crude odds ratios, *Adjusted OR* odds ratios adjusted for sociodemographic variables, anxiety and depressive symptoms, *CI* confidence Interval

## Discussion

### Prevalence of headache trigger factors

The preceding analysis has shown that almost all patients (98.28 %) provided at least one positive response when asked to choose from a predetermined list of trigger factors and the majority reported multiple triggers. At least two triggers were reported by 95.70 % of our sample with similar percentages among the three study groups. Kelman (94.60 %), Fukui et al. (95.50 %), and Baldacci et al. (100 %) presented almost the same results, for migraine. The trigger prevalence in TTH was presented with a percentage of 54.90 % in an epidemiological study in Croatia by Zinadinov et al. and 64.20 % in a recent study in China by Wang et al. [8, 13, 14, 19, 26].

Exposure to triggers does not always lead to a headache onset, even in the same sensitized person. In the current study, it is evident that the attacks are precipitated circumstantially by 76 %, whereas constantly only by 24 %. Our data were similar with previous studies, which support the occasional trigger—induced pattern. It is hypothesized that the potential factors may (1) not act alone but interact in combination with each other and, thus, increase the likelihood of triggering a specific attack [27] (2) have different duration and intensity threshold for headache activation or (3) correlate with patients’ natural history of headache with consequent change in their occurrence [10, 15].

### Trigger factors comparison among headache subtypes

A statistically significant higher median number of possible triggers per patient was recorded in subjects with MA compared with the other headache groups. This comes in accordance with Zivadinov et al. study but not with Wober’s et al. who found similar numbers between migraine and TTH groups [15, 19]. Furthermore, 11 individual triggers showed statistically significant association with MA. Among them, sleep, hormonal variations in women, stress and specific environmental triggers seemed to play a more important role in this subgroup. Some of these triggers were similarly reported in previous studies [8, 11, 16].

The observed predominance of MA may have its origin on the different pathophysiological mechanism of headache subgroups. Previous literature associates MA with cortical spreading depression (CSD), a transient neuronal depolarization wave that spreads across the cerebral cortex and is followed by brain activity inhibition [28, 29]. No signs of CSD in the studies with regional cerebral flow were demonstrated in migraine without aura [30] and in TTH [31]. Various trigger factors, internal or external, may have an enhanced interrelation with CSD as hypothesized by Welch and Chakravarty, who suggested that the triggers affect an already hyper-excitable and susceptible migraine cortex and provoke the onset of CSD due to a concept similar to epileptic seizure pattern [32, 33]. CSD is proposed to activate the cascade of headache pain pathway [34]. Extensive research in this area is needed to clarify the underlying mechanism of these subgroups.

### Most important trigger factors analysis

#### Stressful events and emotional upset

The majority of the literature in the field of headache precipitants demonstrates stress and emotional upset as the main triggers of a headache attack [9, 10, 35]. Supporting this argument, the present study found that stressful life events and intense emotions are the commonest triggers in the whole sample, in both genders and among the three headache subtypes. The relationship between stress and headache crisis is complex. It is proposed that acute stress may affect the biological modulatory pathways leading to increased sensitivity of the migraine cortex [36] nevertheless, there is a lack of experimental evidence. Furthermore, Shoonman et al. failed to find evidence to connect the objective changes in stress-related measured parameters, such as cortisol, to a migraine attack, despite the fact that patients mentioned subjective stress before the headache onset [37]. Perhaps, the perceived stress predominance in many studies can be explained by the findings of Houle et al. who detected a cumulative effect of combined high stress levels and low sleep duration that influences the headache activity, thus, suggesting that stress acts as an enhanced trigger when it interacts with another factor [38]. On the other hand, prodromal symptoms of the migraine process that occur up to 24 h before the attack, especially those related to mood changes such as depressive symptoms or symptoms of hypomania, may be confused with real trigger factors leading to an over-reporting of emotional triggers.

#### Female hormonal variance

Wober et al. emphasized that menstruation is the most important risk factor for the onset and the persistence of headache and migraine attack in the PAMINA study [39]. In this study, hormonal triggers like menstrual and premenstrual period seem to be important, respectively reported by 56 and 42 % of the sample respectively, while similar percentages supporting this argument were found in previous studies [13, 40]. The current study confirmed that MA is more related to female hormones than MoA or TTH in contrast to a population-based study in Croatia [19] and to Macgregor’ study, who linked MoA with estrogen ‘withdrawal’ and MA with high estrogen states [41]. The substantial mechanism between headaches and menses has been widely analyzed and the previous literature has suggested that the fluctuation of serum estradiol and progesterone levels in menstrual cycle is associated with higher headache activity in female migraineurs [42, 43]. Moreover, it has been reported that TTH is not influenced by female hormones variance in such an important way, though it can trigger some attacks [10].

#### Sleep

Regarding sleep disturbances, almost half of our sample identified lack of sleep as an important initiating factor, while oversleeping was 4 times more likely in MA than in MoA and TTH. Changes in sleep hours, including night shift work with irregular shifts between nocturnal and diurnal schedule, appeared to be a strong trigger similar to Ho’s findings, which suggest that individuals who performed shift work had more often headaches [51]. Previous research presented an intermediated relationship between sleep and headaches. According to Kelman et al., short sleep leads to increased severity and frequency of migraine attacks [44]. TTH also seems to be triggered by sleep time in our patients. That is in agreement with the conclusions of a Japanese study that used electronic diaries to record sleep patterns in relation to headache frequency and intensity [45].

#### Fatigue

Fatigue was one of the few precipitating factors cited more in TTH than in the other two study groups, though not statistically significant. Almost half of our subjects reported tiredness as an important trigger, although the physical exhaustion as a headache trigger is questionable due to its common presence as a prodromal symptom of migraine. However, the frequent occurrence of fatigue indicates that its cautious management may contribute to more efficient headache prevention [39].

#### Weather parameters

The statistical analysis of our data showed that weather changes are perceived as a trigger by a 46.55 % of our sample, similar to Robbins [9] but less significant compared to higher percentages of other studies [15, 40]. The discrepancies in this field are probably due to different regional climatic conditions. Previous diary-based publications using meteorological data suggested that more patients believed that weather was a trigger than it actually was [46]. Nevertheless, a group of subjects are sensitive to specific weather variables [47]. Moreover, in the current sample, hot or cold weather, wind, and bright sunlight appeared to be significant important trigger factors to MA patients. The latter one is repeatedly associated with MA [8, 18, 35, 48].

#### Nutrition

Food did not seem to be a frequent trigger for headaches in our study. Hunger and skipping meals were more common, reported in percentages around 30 % in contrast to other studies, where higher numbers were demonstrated [13, 16, 49]. Cultural and social habits of different populations may explain the detected differentiation.

### Anxiety and depression in headache patients: association with triggers recording

It is important to control anxiety and depression comorbidity in studies with pain [22]. The psychological status of the patients is considered to be tightly associated with headaches due to their influential role on the headache prognosis [23].

In the current study, HADS was applied for two main reasons. Firstly, we wanted to investigate the prevalence of anxiety and depression in the three study groups and, secondly we wanted to test the hypothesis that the likelihood of trigger reporting would be independent of the presence of anxiety or depression, as the latter may have a confounding effect. In our study, we found large numbers of anxiety symptoms and a slightly lower number of depressive symptoms in headache patients but no statistically significant differences among the subgroups in accordance to the Zwart et al. study, which showed no dependency between anxiety and depression disorders and diagnostic headache category [50].

Adjustment for the sociodemographic characteristics and psychological symptoms did not affect the association between the type of headache and trigger factors, suggesting that the number of triggers factors reported in our study is independent of these variables.

### Limitations and strengths of the study

This study was based entirely on patients from a single tertiary clinic and therefore the results cannot be applied to other patients from different settings (for example primary care) or to the general population. In addition, the sample was relatively small and predominantly female and power may have been undermined. The possibility of a recall bias should be considered due to the retrospective nature of asking about the triggers. In addition, we have asked a large number of headache triggers and it is likely that some patients may have over-reported the likelihood of a given trigger to provoke an attack.

The main strengths were that all patients were interviewed by the same clinician; none of the patients were receiving preventive medication; the data of the three groups were comparable; and the diagnoses were precisely based on the revised criteria of ICD-III Beta.

## Conclusion

The current study gives a general picture of the headache trigger factors in Athens, Greece. Our data showed that migraine and TTH patients reported multiple triggers, on a frequent but not consistent basis, independent of their anxiety or depressive symptoms. Stressful events and emotional upset were the most cited triggers regardless of the gender or the headache subtype. MA predominantly demonstrated the highest frequencies in almost all triggers, being significantly different from MoA and TTH in one third of them. Future studies should focus on the whole context that surrounds the trigger and the probable trigger synergy in headache precipitation, as these could help in the prevention and management of these disabling conditions.

## Abbreviations

MoA: migraine without aura
MA: migraine with aura
TTH: tension type headache
HADS: Hospital Anxiety and Depression Scale
